# Social density processes regulate the functioning and performance of foraging human teams

**DOI:** 10.1038/srep18260

**Published:** 2015-12-17

**Authors:** Andrew J. King, Julia P. Myatt, Ines Fürtbauer, Nathan Oesch, Robin I. M. Dunbar, Seirian Sumner, James R. Usherwood, Stephen Hailes, M. Rowan Brown

**Affiliations:** 1 Biosciences, College of Science, Swansea University, Singleton Park, Swansea, SA2 8PP, UK; 2 Biosciences, University of Birmingham, Edgbaston, Birmingham, West Midlands, B15 2TT, UK; 3 SENRG, Department of Experimental Psychology, South Parks Road, Oxford, OX1 3UD, UK; 4 School of Biological Sciences, University of Bristol, Woodland Road, Bristol BS8 1UG, UK; 5 Structure & Motion Laboratory, Royal Veterinary College, Hatfield, Hertfordshire, AL9 7TA, UK; 6 Department of Computer Science, University College London, Gower Street, London WC1E 6BT, UK; 7 College of Engineering, Swansea University, Bay Campus, Fabian Way, Swansea SA1 8EN

## Abstract

Social density processes impact the activity and order of collective behaviours in a variety of biological systems. Much effort has been devoted to understanding how density of people affects collective human motion in the context of pedestrian flows. However, there is a distinct lack of empirical data investigating the effects of social density on human behaviour in cooperative contexts. Here, we examine the functioning and performance of human teams in a central-place foraging arena using high-resolution GPS data. We show that team functioning (level of coordination) is greatest at intermediate social densities, but contrary to our expectations, increased coordination at intermediate densities did not translate into improved collective foraging performance, and foraging accuracy was equivalent across our density treatments. We suggest that this is likely a consequence of foragers relying upon visual channels (local information) to achieve coordination but relying upon auditory channels (global information) to maximise foraging returns. These findings provide new insights for the development of more sophisticated models of human collective behaviour that consider different networks for communication (e.g. visual and vocal) that have the potential to operate simultaneously in cooperative contexts.

Models of collective animal behaviour in which every agent updates its trajectory based on the trajectories of its neighbours find that with an increasing density of agents, the system (i.e. the swarm, school, flock, or crowd) switches from a state of disordered movement to a state of coherent collective movement^1^,^2^. One of the best-known and studied examples is pedestrian flow of human crowds. Models and empirical data^3^,^4^,^5^ show that at low densities, pedestrians tend to move freely, and the behaviour of the crowd can be partially compared with the behaviour of gases. At intermediate densities, crowd motion becomes highly coordinated and ‘lanes’ of bidirectional flow develop spontaneously. At high densities, coordinated motion can break down and crowds typically show stop-and-go waves and crowd turbulence. Whilst much effort has been devoted to describing and predicting collective human motion in the context of pedestrian flows^6^, there is a distinct lack of empirical data investigating the effects of social density on human behaviour in other relevant contexts^7^,^8^.

A fundamental human behaviour that is often dependent upon collective motion is foraging. In modern-day traditional societies, and in our evolutionary past, individuals have benefited from coordinating their movements and making collective decisions about how to obtain resources; searching for dispersed food sources in a patchy, uncertain environment, and then returning to a central location with this food resource^9^,^10^,^11^. Indeed, individual search costs can be reduced, and group performance improved, if a number of foragers cooperate by coordinating their behaviour and exchanging information about encountered food items^12^,^13^,^14^. Despite the importance of coordinated behaviour in a foraging context, we know very little about what factors predict the success or failure of such groups. Such knowledge may have important consequences for understanding the functioning of modern day teams and organisations^15^,^16^.

Given the importance of social density on the state and order of collective behaviours in a variety of non-human and human systems^17^,^18^,^19^, we test the hypothesis that social density processes similarly regulate the functioning and performance of human teams in a cooperative foraging task^20^,^21^ (Fig. 1). In previous work, King *et al.*^8^ devised a simple social foraging paradigm where people foraged in a patchy and uncertain environments. They showed that communication (and especially local communication via gesticulations) is a crucial aspect of the organisation of social coordination in small groups of people. Here, we used this setup to test a series of predictions with respect to both the functioning and performance of human teams at different social densities. If we assume random interactions among team members in a foraging arena of constant size, then the total number of interactions among *n* team members would increase as *n*(*n* − 1). We use this simple paradigm to explore the functioning and performance of teams at low (N = 4), intermediate (N = 8, 12) and high (N = 24) social densities. By design, these group sizes are also similar to those preferred by (or which emerge in) modern societies and hunter-gatherer groups^22^,^23^,^24^. These discrete (sub)grouping patterns are also thought to reflect hierarchical processing of social information in humans^25^, and we therefore we assume our density treatments to represent ecologically relevant group sizes.

First, we explore team functioning. Often, in biological systems, coordination in the motion of individuals can have important fitness consequences, whereby more coordinated individuals can more effectively detect and respond to threats or potential resources^16^,^26^,^27^,^28^,^29^. Therefore we explore the coordination of foragers’ movements during foraging as a measure of team ‘functioning’. We expected that at low forager density (N = 4) individuals would interact with one another at low rates owing to the potentially large inter-individual distances^20^, providing little opportunity for information transfer via local communication^21^ and therefore show low coordination in their movements. As forager densities increase (N = 8, 12) we expected this to afford higher rates of social interaction which would facilitate local information transfer and coordination among foragers^30^,^31^. At high forager densities (N = 24), however, we expected coordination to break down as a consequence of congestion effects which may inhibit local information transfer^32^,^33^.

If the functioning of teams does change as a consequence of forager density, then we also expected that this would have consequences for team performance, and predicted intermediate densities to perform better for at least three interconnected reasons. First, the speed and accuracy of decision-making is predicted to be highest where there is opportunity for social information transfer^34^,^35^, and we expect local information transfer to be greater at intermediate densities (see above). Second, intermediate densities represent relatively larger teams that are predicted to outperform smaller teams in terms of decision-making accuracy as a consequence of wisdom-of-crowd effects^36^,^37^ whereby the aggregation of information across multiple individuals (pooling information) can result in decisions that are often better than the ones that could have been made by any single individual^36^,^38^. However, this information-pooling benefit of larger groups is predicted to diminish as group size exceeds more than 20 individuals^39^. Third, sports teams tend to be 12 ± 3 individuals because of assumed limits on individuals’ capacities to engage with each other (i.e., “sympathise”), and hence their ability to coordinate^40^. Thus, we expected that opportunity for, and quality of, information-sharing to be maximised at intermediate densities.

## Material and Methods

### Subjects

Visitors to the Royal Veterinary College Open Day on 7^th^ May 2011 were invited to take part in our experiments. 144 English speakers (111 females, 33 males) aged 16–54 signed up prior to data collection, and were randomly assigned to mixed-sex team sizes of N = 4, 8, 12 and 24, since group sex ratios were not found to significantly alter foraging performance in small teams undertaking the same experiment^8^. Teams of 4, 8, 12, and 24 took part in the foraging task simultaneously at three different time slots throughout the day.

### Experiments

**Figure 1:**
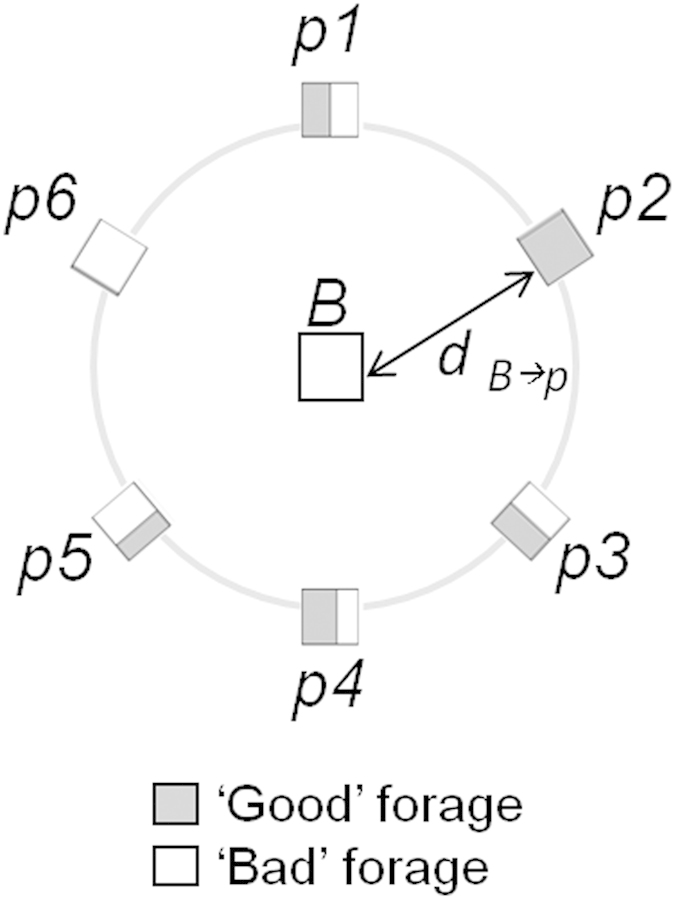
Foraging arena. The arena consisted of a circle (diameter 10 m) with a home base (*B*) at the centre and six foraging patches (squares *p1*, ..., *p6*) at the edge, arranged every 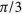 radians. The distance between *B* and any patch (*d*_*B→p*_) and between neighbouring patches (e.g. *d*_*p1→ p2*_) was 5 m. The shaded portions of the foraging patches represent good forage tokens, and the un-shaded portion bad forage tokens. The position of patches was randomised across experiments.

Experiments were conducted outdoors in four circular ‘foraging arenas’ which contained six foraging patches and a ‘home base’, following King *et al.*^8^ (Fig. 1). Participants could not see inside the home base or any of the foraging patches and had to put their hand in and pull out a token to see whether it was ‘good’ or not. The foraging patches contained a mix of 300 ‘good’ (green) and ‘bad’ (purple) tokens and varied in quality from 5, 35, 50, 65 to 95% good tokens. The location of patches was randomised across experiments. Prior to the experiment informed consent was obtained from all subjects and participants were given instructions (by AJK) to collect as many green tokens as possible, and as few purple tokens as possible; this ensures people attempt to find and utilise the best foraging patches and not just the number, or proportion, of green tokens^8^. There were no restrictions on visits to the patches, but only one token could be collected at a time and every token collected had to be deposited in the home base. All participants were instructed to walk. The experiment was started by a whistle blow. If any rules were broken during the experiment (e.g. carrying multiple tokens, running) the whistle was blown again by the instructor, who was standing at the edge of the arena. Participants were not aware of exactly how long the task would last, but knew that the whole process would not take more than 30 minutes. In reality, all trials lasted for a period of 10 minutes. Individuals were given an incentive to contribute to the group’s foraging score through competing with other groups participating on the day, and were told that their performance would be compared to other groups. Scores were later revealed online, enabling participants to keep track of how their group performed. All experiments described here were approved by the Royal Veterinary College ethics committee, and performed in accordance with The British Psychological Society guidelines and regulations for conducting research with human participants.

### Data collection

Participants wore baseball caps carrying data loggers that comprised a microcontroller and a rechargeable 2,200 mAh lithium polymer battery which powered a GPS antennae and module that recorded single frequency L1 raw range data (uBlox LEA-4T GPS module). All data were collected at 1 Hz and stored on a micro-SD card. The same data-logger design with similar sampling regimes is described in Haddadi *et al.*^41^ and King *et al.*^27^. All GPS devices were time synchronised to UTS, providing the position vector, 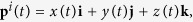 for each individual (*i*), for the duration (T) of the experiment T = {0, τ, 2τ, …, 600τ}, τ = 1 second. These GPS data were used to explore the functioning and performance of teams as described below.

### Social density

Inter-individual forager distances were calculated across all dyads, for every second (τ) so that we could explore the variability in inter-individual distances (the range) and the most frequent distance between participants, 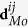 (Equation 1) as:


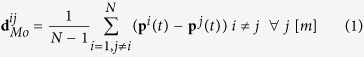


### Team functioning

To examine functioning, we use the degree of coordination within teams, calculated as the velocity cross-correlation^42^,^43^ *C*, between individuals *i* and *j* in time (Equation 2):


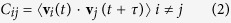


Velocity cross-correlation is typically used to identify leader-follower dynamics within groups^42^. Here, we calculate the mean velocity cross-correlation across all dyads 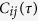 for *τ* between −7 and +7 seconds. We chose this time period since foragers took a mean ± S.D. 7 ± 0.4 seconds to move between any foraging patch and the home base, and thus provides a coarse measure of how correlated the motion of the whole team was over a time period that is equivalent to one inward or outward movement. High mean values of 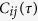 are thus indicative of coordinated motion between foraging patches and the home base (i.e. groups with dyads that typically are travelling with similar acceleration and direction), whilst low mean values of 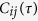 would suggest individuals move independently and do not tend to follow one another’s movements.

### Performance of teams

We calculated the distance travelled by an individual,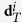 over the course of the experiment (Equation 3) as:


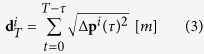


where 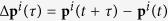. We also calculated the time (s) that each person took to move to and from *B*, at the centre, and one of the six foraging patches *p1*, …, *p6* at the edge of the foraging arena. These measures gave us a measure of how quickly foragers were able to move towards and away from the available foraging patches.

To investigate accuracy of foraging decisions, we measured the accuracy of the teams foraging at the end of experiments, counting the total proportion of good forage deposited in the home base at the end of experiments. We also calculated the performance of teams over time, by summing the product of the number of visits to each patch times the patch-specific probability of a good counter being taken. To do this, the foraging arena was divided into elements of 1 m^2^ and the frequency (density) of visits by group members was deduced over 1-minute intervals.

### Statistical Analyses

To compare the distance travelled and time taken to complete a return trip (i.e. collect and deposit forage), we used a Linear Mixed Model in R version 3.1^44^ and fitted team size (N = 4, 8, 12 or 24) as a fixed effect, and group identity (a-l) as a random effect (thus controlling for the non-independence of foragers movements in the same group). We compared means for: (i) the total number of forage items collected by teams of different size, (ii) the accuracy of foraging collected (good forage/total forage), and (iii) values of 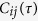 using a one-way Analysis of Variance (ANOVA) and Tukey’s range test for post-hoc analysis in SPSS^45^ (each dependent variable was normally distributed as indicated by Shapiro-Wilk tests).

## Results

### Social density

**Figure 2:**
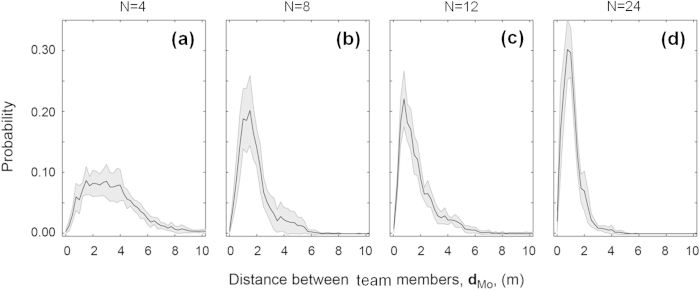
Social density. Probability distributions (black line) and standard deviation (grey) associated with the most frequent inter-individual forager distance (metres), 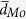, across ten 1-minute bins, for teams of (**a**) N = 4, (**b**) N = 8, (**c**) N = 12, and (**d**) N = 24. The most frequent 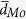 for N = 4, 8, 12, 24 is 3.0, 1.75, 1.65, 0.95 m respectively.

At low density (N = 4) inter-individual forager distances were highly variable, with individuals typically observed at distances between 1.5 and 4 m (Fig. 2a). At high density (N = 24), inter-individual distances were more consistent, and tended to be just 0.95 m (Fig. 2b). Intermediate densities (N = 8 and N = 12), in contrast, showed the most frequent inter-individual distances at 1.65 m and 1.75 m respectively (Fig. 2c,d).

### Functioning

**Figure 3:**
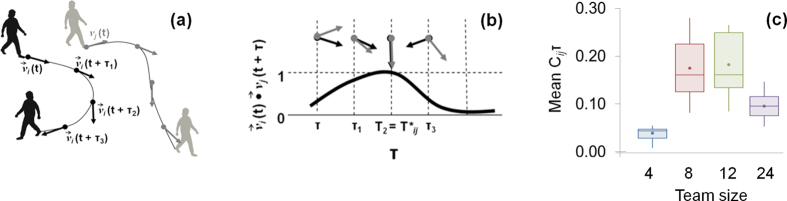
Forager coordination. (**a**) Schematic diagram illustrating correlated velocity between two individuals 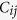 at time intervals *τ*. In this example. 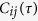 is maximised at 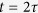 where speed and direction of individuals *i* and *j* are most coordinated. (**b**) Graphical illustration of the correlated velocity of individuals *i* and *j* as shown in schematic in (**a**). Illustrations in (**a**,**b**) are inspired by those presented in Nagy *et al.*^42^ (**c**) Box and whisker plot illustrating range (whiskers), interquartile range (box), median (line) and mean (point) 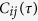 for teams of N = 4 (blue), N = 8 (red), N = 12 (green) and N = 24 (purple) for *τ* between −7 and +7 seconds. 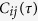 is maximised between −1 and +1 seconds for N = 8 and N = 12 (upper part of whisker), whilst 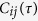 is low for N = 4 and N = 24 for this period.

Coordination, measured as mean values of 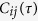 (Fig. 3a,b), differed according to density treatment (ANOVA: 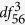, F = 35.98, *P* < 0.001), and post-hoc tests indicated that teams at intermediate forager densities (N = 8, N = 12) showed higher levels of coordination than teams at low (n = 4) or high (n = 24) densities over the course of the experiments (Fig. 3c). Variation and maximum values of 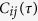 are also instructive; N = 8 and N = 12 showed largest variation (Fig. 3c) and maximum cross-correlations were observed for *τ* between −1 and +1, while teams of N = 4 and N = 24 showed less variation (Fig. 3c) and low cross-correlations for *τ* between −1 and +1. This suggests that individuals in teams of N = 8 and N = 12 were more likely to respond to each other’s movements, and did so especially quickly.

### Performance

**Figure 4:**
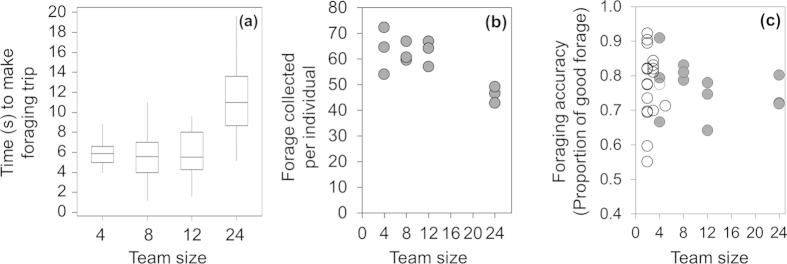
Foraging effort and performance. (**a**) Box and whisker plot illustrating range (whiskers), inter-quartile range (box), and median (line) time taken for individuals in teams of N = 4, 8, 12, and 24 to complete a foraging trip between the home base and a foraging patch. (**b**) Mean number of items of forage collected per individual as a function of team size. (**c**) Mean foraging accuracy at the end of experiments (total proportion of good forage collected) for teams of 4, 8 12 and 24 individuals (grey circles, this experiment). Data also shown for teams of 2–5 individuals (open circles) for 20 teams taken from King *et al.*^8^ collected using the same foraging paradigm.

**Figure 5:**
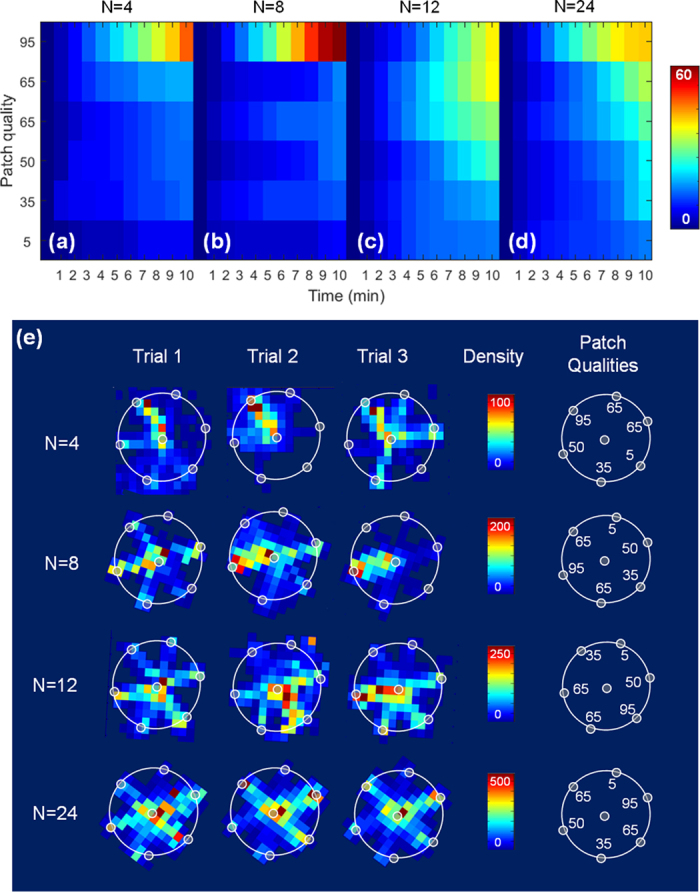
Forager decisions and accuracy. (**a–d**) show heat maps indicating the mean cumulative foraging visits by individuals to patches of different quality (y-axis) as a function of time with 1-minute bins (x-axis) in teams of N = 4, 8, 12 and 24 respectively. The colour bar represents the number of visits per individual forager, with warmer colours representing more visits. (**e**) The first three columns show the foraging arenas (large white circles) and foraging patches (small white circles at the arena edge) for three replicates at different social densities (rows). Each foraging arena has been divided into 1 m^2^ elements and the colour of each element is the frequency of visitation by group members over the course of the experiment. The colour bar indicates the element frequency, with warmer colours representing a higher frequency of forager visits. The locations of foraging patches were randomly assigned for each team size, and are indicated by the schematic arena maps “patch qualities” on the right-hand side.

Since inter-individual distances in N = 24 teams tended to be just 0.95 m, individuals experienced congestion effects that resulted in slower foraging trips; the time taken to move from the home base to an outer foraging patch and back was significantly slower in N = 24 teams compared to teams at other densities (N = 24 vs 12: LMM, Effect = −2.51, SE = 0.72, t = −3.50, *P* = 0.009; N = 24 vs 8: LMM, Effect −2.67, SE 0.73, t = −3.67, *P* = 0.006; N = 24 vs 4: LMM, Effect = −2.61, SE = 0.79, t = −3.31, *P* = 0.007). Teams of N = 4, 8, and 12 were not statistically different from each other in the time they took to move between patches and the home base (Fig. 4a). Distance travelled by teams was comparable across density treatments (N = 24 vs 12: LMM, Effect = −0.65, SE = 0.37, t = −1.78, *P* = 0.078; N = 24 vs 8: LMM, Effect 0.35, SE 0.40, t = 0.88, *P* = 0.383; N = 24 vs 4: LMM, Effect = −0.30, SE = 0.57, t = −0.54, *P* = 0.592). This suggests that N = 24 teams did not alter their movement paths between patches but did walk more slowly, resulting in the total amount of forage collected by individuals in N = 24 teams being significantly less than teams of other sizes (Fig. 4b; ANOVA, 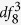, F = 6.18, *P* = 0.018; Tukey’s tests: N = 24 vs. 4, *P* = 0.026; N = 24 vs. 8, *P* = 0.038; N = 24 vs. 12, *P* = 0.035). However, despite increased coordination at intermediate densities, and reduced total forage collected at the largest density treatment, team foraging accuracy was equivalent across our density treatments (ANOVA: 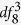, F = 0.82, *P* = 0.517; Fig. 4c), and all teams converged on the best foraging patches half-way through the experiment (Fig. 5a–d), with N = 12 and N = 24 teams distributing their foraging effort across the best (95%) and next best (65%) quality patches (Fig. 5c,d).

## Discussion

Our investigation into the functioning and performance of human teams in a central-place foraging arena, using high-resolution GPS data, has shown that team functioning (level of coordination in motion) is maximised at intermediate social densities, as predicted. Classic field experiments have shown that people tend to respond to the gaze direction of others, with the strength of response increasing with the number of other people already gazing^46^ and more recent work reveals that visual interactions between pedestrians occur primarily within a 2 m range^47^. In our experiments, foragers in intermediate density teams tended to be 1.65–1.75 m from one another, whilst foragers in low or high social density teams (N = 4, 24) were generally more than 3 m or less than 1 m from each other, respectively. These differences in inter-individual distances could have therefore been constraining visual channels (local information) at low densities (N = 4) and inhibiting it at high densities (N = 24), and this may offer an explanation for the differences in coordination we observed across our density treatments.

Coordinated patterns of behaviour in many group-living animals afford fast and accurate collective decisions where all (or the majority of) individuals adopt the same choice^48^,^49^, with coordination in mobile animal groups (of both predators and prey) having important fitness consequences^26^,^27^,^50^. Here though, coordinated motion when moving around the foraging arena had little consequence for the performance of teams since all teams showed similar levels of accuracy in foraging (Fig. 4c). In fact, it appears that being coordinated in motion when moving around the arena did not reflect either foraging accuracy (Fig. 4c) or the way in which teams used patches (Fig. 5a), with teams of N = 12, N = 24 tending to distribute their foraging efforts across the best (95%) and next best (65%) patches and N = 4 and N = 8 teams concentrating on the single best patch. Thus, whilst teams showed differing levels of coordination in motion, they all coordinated their decisions to relevant food patches. We believe that this may occur as a result of foragers’ ability to exchange information about the best foraging patches independent of their visual interaction ranges (i.e., via verbal communication). Indeed, earlier experiments by King *et al.*^8^ using the same set-up with smaller groups (N = 2–5) found that conversation was constant throughout experiments, suggesting verbal communication may allow information exchange across many members in a short time frame. These results therefore highlight the importance of considering not only different networks for communication (e.g. visual and vocal), but also different metrics to describe “coordination” of foragers in future works.

Although foraging performance was not statistically different across our different social densities, our data suggest that foraging in larger teams may enable foragers to reduce variance in the proportion of good forage collected^51^. This can occur if the rates at which individuals choose to forage from the best food patch increases with the number of other individuals choosing the patch^46^; this would result in a corresponding decrease in variance in patch choices^51^. Therefore, the higher social density treatments could be experiencing a group-size related benefit whereby their precision of foraging is improved. However, increased replicates of our experiments at larger group sizes would be required to test this hypothesis in a meaningful way.

In addition to quantifying different networks for communication and associated metrics to describe collective dynamics in future works (see above), another important direction for future work is to examine the relationship between functioning and performance of teams in repeated games, rather than the one-shot games studied here. The dynamics of coordination and cooperation can operate differently in repeated games^52^,^53^, and thus the functioning and/or performance of teams may not hold true across repeated interactions^38^. Similarly, we do not yet know how individual forager decisions are integrated and scale to the team performance; there are numerous mechanisms by which individual choices could lead to the team reaching consensus on the best foraging patches^48^. Exploring the variety of different possible decision mechanisms will be important to understanding the mismatch between coordination and performance we observe here. Such insight will be key because it will pin-down the extent to which experiments like these help us understand the constraints on coordination/cooperation in human groups in other ecologically relevant tasks, for example, resource or predator defence^54^,^55^,^56^. As such, we anticipate that our findings will inform the development of more sophisticated models of human collective behaviour that consider different communication networks that may operate simultaneously in cooperative contexts.

In summary, our experimental approach has produced quantitative and qualitative insights about how social density affects human behaviour in a cooperative context. We have used high resolution GPS tracking and spatial-temporal analysis to quantify a number of aspects of human team functioning and performance, and our findings suggest foragers rely upon visual channels (local information) to achieve coordination but use auditory channels (global information) to maximise foraging returns. We hope that these measurements on the dynamics of team behaviour in this experimental setting can inspire the development of more sophisticated experiments and models to understand human collective behaviour and psychological mechanisms underpinning such collective action which will have important consequences for understanding the functioning of modern day teams and organisations.

## Additional Information

**How to cite this article**: King, A. J. *et al.* Social density processes regulate the functioning and performance of foraging human teams. *Sci. Rep.* **5**, 18260; doi: 10.1038/srep18260 (2015).
